# Extramacular paracentral acute middle maculopathy-like retinal ischemia after vitrectomy for epiretinal membrane^☆^

**DOI:** 10.1016/j.ajoc.2024.102221

**Published:** 2024-11-14

**Authors:** Chihiro Koiwa, Pingyu Chi, Shutaro Yamamoto, Shintaro Nakao

**Affiliations:** Department of Ophthalmology, Juntendo University School of Medicine, Tokyo, Japan

**Keywords:** Arteriosclerosis, Local anesthesia, Paracentral scotoma

## Abstract

**Purpose:**

To describe two cases of extramacular paracentral acute middle maculopathy (PAMM)-like retinal ischemia after vitrectomy with internal limiting membrane (ILM) peeling for epiretinal membrane (ERM).

**Observations:**

Case 1 involved a 78-year-old woman with a history of hyperlipidemia and preoperative visual acuity (VA) of 20/20. Case 2 involved a 72-year-old man with a history of hypertension and hyperlipidemia, and preoperative VA of 20/32. In both cases, cataract surgery and vitrectomy with ILM peeling were performed for the unilateral ERM and cataract. On postoperative day 1, retinal whitening was observed at separate extramacular areas from ILM peeling in the macular region in both cases. Optical coherence tomography revealed a high-intensity reflection in the inner nuclear layer, and optical coherence tomography angiography showed a perfusion defect in the deep capillary plexus (DCP), which corresponded to the area of retinal whitening. Fluorescein angiography revealed no significant abnormalities, leading to a diagnosis of PAMM-like retinal ischemia. In Case 1, retinal whitening disappeared at postoperative week 2, and the corrected VA improved to 20/20 at postoperative month (POM) 6. In Case 2, retinal whitening resolved at POM 1 and POM 4.5, and VA improved to 20/16. However, both cases showed residual perfusion defects in some deep retinal vessels, accompanied by scotomas corresponding to these areas.

**Conclusions and importance:**

We observed two cases of extramacular PAMM-like retinal ischemia that developed after vitrectomy with ILM peeling. Although both cases showed improved VA, blood flow disturbances at the DCP level and associated visual field defects persisted.

## 1 Introduction

Paracentral acute middle maculopathy (PAMM) was first described by Sarraf et al., in 2013.^1^ Optical coherence tomography (OCT) of PAMM reveals a hyperreflective band in the macula that is confined to the level of the inner nuclear layer (INL).^2^ Christenbury et al. were the first to report that OCT angiography (OCTA) confirmed the presence of ischemia in the deep retinal capillary plexus (DCP) in PAMM associated with central retinal artery occlusion.^3^ Non-surgical cases such as sickle cell crisis, Purtscher's retinopathy, and inflammatory occlusive retinal vasculitis have been reported to be associated with PAMM.^4^ Post-surgical cases of PAMM have been documented following cataract surgery,^5^ proliferative diabetic retinopathy surgery,^6^ and pterygium.^7^ However, only two cases of PAMM have been reported following epiretinal membrane (ERM) surgery with internal limiting membrane (ILM) peeling.^8^^,^^9^ Herein, we describe two cases of PAMM-like retinal ischemia after vitrectomy for ERM. Unlike the two previously observed cases, in our patients, lesions were observed in extramacular areas separate from the site of ILM peeling.

## 2 Case reports

**Fig. 1:**
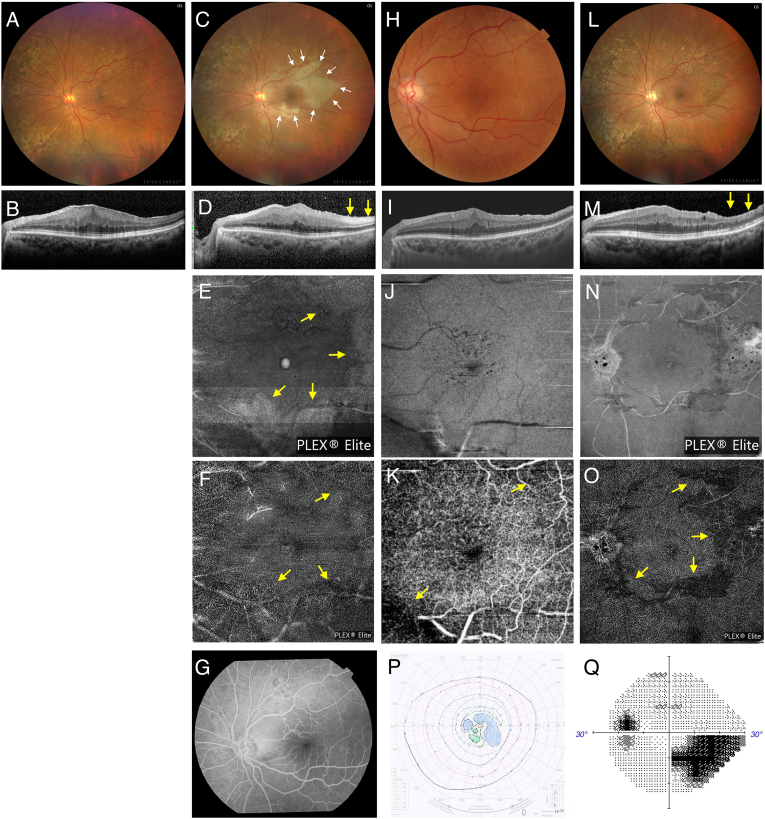
Case 1 (78-year-old woman) A) Color fundus photograph and B) optical coherence tomography (OCT) showing epiretinal retinal membrane (ERM) in the left eye. C) On postoperative day (POD) 1, retinal whitening is observed beyond the internal limiting membrane (ILM) peeling area (white arrows), and D) OCT reveals high-intensity reflection in inner nuclear layer (INL) (yellow arrow). E) En face OCT (6 × 6 mm^2^ scan) shows high-intensity changes in the same area (yellow arrows), and F) OCT angiography (OCTA) at the deep capillary plexus (DCP) level shows reduction in blood flow (yellow arrows), G) Fluorescein angiography appears nearly normal. H-J) The retinal whitening, high-intensity reflection in INL on OCT, and high-intensity changes on en face OCT has resolved at postoperative week (POW) 2; K) however, blood flow abnormalities at the DCP level remains detectable on OCTA (yellow arrows). L) Fundus photograph at postoperative month (POM) 6 shows nearly normal findings. M) On OCT, thinning of the INL is observed (yellow arrow). N) High-intensity changes on en face OCT (12 × 12 mm^2^ scan) has improved. O) By POM 6, blood flow impairment persists on OCTA at the DCP level (yellow arrows). P) On POD 1, a paracentral scotoma consistent with the ischemic area is observed, which has partially improved but persists at POM 6 (Q).

Case 1: A 78-year-old woman presented to our hospital for the surgical treatment of ERM and cataract in her left eye (Fig. 1A and B). The patient's medical history included hyperlipidemia. Best-corrected visual acuity (BCVA) was 20/20 in the left eye, and intraocular pressure was within normal limits. No abnormalities were observed in the anterior segment; however, cataract and ERM were observed. A 25-gauge vitrectomy (Alcon Constellation Vision System; Alcon Surgical, Fort Worth, TX, USA) with ILM peeling and cataract surgery was performed due to her complaint of metamorphopsia in the left eye. No additional hyaloid membrane removal was performed outside the area of ILM peeling. The ILM was stained using Brilliant Blue G and a flap was created using a diamond-dusted membrane scraper; following this, the ILM was peeled using the Alcon Grieshaber Revolution DSP ILM forceps (Alcon Surgical, Fort Worth, TX, USA). For local anesthesia, 3 mL of 1 % xylocaine was administered sub-Tenon. The intraoperative infusion pressure was set at 30 mmHg and the procedure lasted for 30 min. On postoperative day (POD) 1, retinal whitening beyond the ILM peeling area was observed in the macular region and the extramacular area (Fig. 1C). OCT (Spectralis system; Heidelberg Engineering, Heidelberg, Germany or Topcon DRI OCT Triton plus, Topcon Corporation, Tokyo, Japan) revealed high-intensity reflection in the INL (Fig. 1D). En face OCT (PLEX Elite 9000; Carl Zeiss Meditec, Dublin, CA, USA or Topcon DRI OCT Triton plus; Topcon Corporation, Tokyo, Japan) showed high-intensity changes in the same area (Fig. 1E). OCTA (PLEX Elite 9000, Carl Zeiss Meditec, Dublin, CA, USA, or Topcon DRI OCT Triton plus; Topcon Corporation, Tokyo, Japan) at the superficial capillary plexus (SCP) level and fluorescein angiography (FA) appeared nearly normal; however, DCP-level retinal perfusion defects were observed (Fig. 1F and G). Retinal whitening and high-intensity changes on en face OCT resolved at postoperative week (POW) 2 (Fig. 1H–K); however, blood flow abnormalities at the DCP level remained detectable on OCTA at postoperative month (POM) 6 (Fig. 1L–O). BCVA decreased to 20/200 on POD 1, and a paracentral scotoma consistent with an ischemic area was observed (Fig. 1P). By POM 6, the BCVA had improved to 20/20, but the paracentral scotoma persisted (Fig. 1Q).

**Fig. 2:**
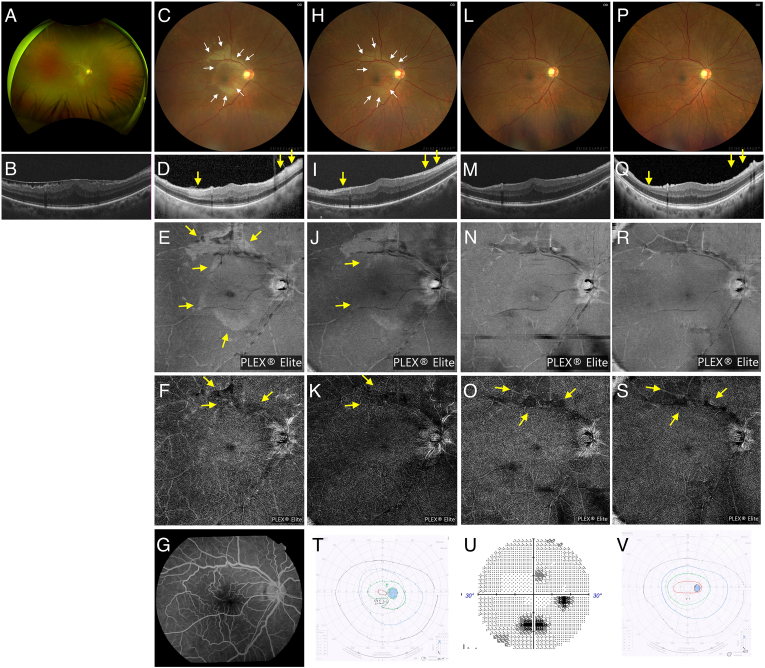
Case 2 (72-year-old man) A) Color fundus photograph and B) optical coherence tomography OCT showing epiretinal membrane (ERM) in the right eye. C) On postoperative day (POD) 1, retinal whitening is observed beyond the internal limiting membrane (ILM) peeling area (white arrows), and D) OCT reveals high-intensity reflection in INL (yellow arrow). E) On POD 3, en face OCT (12 × 12 mm^2^ scan) shows high-intensity changes in the same area (yellow arrows), and F) OCT angiography (OCTA) at the deep capillary plexus (DCP) level shows reduction in blood flow (yellow arrows); G) Fluorescein angiography appears nearly normal. (H–K) At postoperative week (POW) 2, the retinal whitening, high-intensity reflection in INL on OCT, and high-intensity changes on en face OCT has partially improved (white and yellow arrows), while reduction in blood flow at the DCP level persisted (yellow arrows). (L–N) At postoperative month (POM) 1, retinal whitening, high-intensity reflection in INL on OCT, and high-intensity changes on en face OCT are completely resolved. (O) However, decreased blood flow at the DCP level persists (yellow arrows). P) Fundus photograph at POM 4.5 shows nearly normal findings. Q) On OCT, thinning of the INL is observed (yellow arrow). R) High-intensity changes on en face OCT has improved; S) however, blood flow impairment persists on OCTA at the DCP level (yellow arrows). T) On POD 1, Goldmann perimetry reveals a visual field defect corresponding to the area of retinal whitening. U) Humphrey visual field analyzer reveals that the paracentral scotoma persisted until POM 4.5; (V) however, it is not detectable by Goldmann perimetry at POM 1.

Case 2: A 72-year-old man presented to our hospital for surgical treatment of ERM and cataract in his right eye (Fig. 2A and B). The patient's medical history included hypertension and hyperlipidemia. BCVA in the right eye was 20/32, and intraocular pressure was within normal limits. The anterior segment showed no abnormalities. The surgical procedure was the same as that employed in Case 1. Local anesthesia was administered using 3 mL of 1 % xylocaine via a transconjunctival retrobulbar injection, and the procedure lasted for 38 min. On POD 1, retinal whitening extending beyond the area of ILM peeling was observed in the extramacular and macular regions (Fig. 2C). OCT revealed high-intensity reflections in the ILM (Fig. 2D). En face OCT revealed high-intensity changes in the same region (Fig. 2E). OCTA at the SCP and FA levels appeared nearly normal (Fig. 2G). High-intensity changes on en face OCT resolved at POM 1 (Fig. 2N), but blood flow abnormalities at the DCP level persisted on OCTA at POM 4.5 (Fig. 2S). The BCVA was 20/50 on POD 1, with paracentral visual field defects (Fig. 2T). By POM 4.5, BCVA had improved to 20/16, but the paracentral scotoma remained (Fig. 2U). Goldmann perimetry at POM 1 suggested an improvement in paracentral scotoma (Fig. 2V); however, Humphrey visual field analyzer at POM 4.5 revealed that these defects persisted (Fig. 2U).

## 3 Discussion

Systemic diseases associated with PAMM include sickle cell crisis, Purtscher's retinopathy, inflammatory occlusive retinal vasculitis, post-H1N1 vaccination, hypertensive retinopathy, migraine disorders, and post-upper respiratory infection.^4^ Furthermore, underlying arterial disease may lead to pre-existing hypoperfusion that may be further compromised by raised intraocular pressure during the procedure or via raised orbital pressure from anesthesia.^5^ The two cases presented here had systemic conditions, such as hypertension and hyperlipidemia, that could lead to arteriosclerosis.

In cases of PAMM following non-vitreoretinal surgery, an incidence rate of 0.068 % after cataract surgery have been reported.^5^ Additionally, cases following pterygium surgery using sub-Tenon's or retrobulbar anesthesia have been documented.^7^ The mechanisms suggested for anesthesia-induced PAMM include vasoconstrictive effects of anesthetics on the retinal arteries, retinal arterial hypoperfusion due to intraocular pressure spikes, and mechanical compression of the optic nerve and central retinal artery caused by anesthetics injected into the orbit. No cases of PAMM following cataract surgery under general or topical anesthesia have been reported.^5^^,^^10^ Therefore, the use of sub-Tenon's or retrobulbar anesthesia may have contributed to the onset of the PAMM-like retinal ischemia in the two cases presented here.

Nakashima et al. reported that PAMM occurred in 3.6 % of patients after a 25-gauge pars plana vitrectomy for proliferative diabetic retinopathy, contributing to the development of multiple factors, including vasoconstriction, intraocular pressure spikes, and embolism.^6^ In surgeries in which the intraocular pressure control system was switched off and a relatively low pressure of 15 mmHg was used, PAMM did not occur (0/75 eyes), whereas cases with pressure set at 25 mmHg showed a higher risk of PAMM (6.6 %, 7/106 eyes). In the present case, the intraoperative infusion pressure was set at 30 mmHg. These findings suggest that in patients at high risk, it may be prudent to consider setting a lower intraocular pressure during surgery. However, no findings suggestive of retinal vascular spasm or occlusion were observed during the surgery. Furthermore, because peripheral vitrectomy under ocular compression was not performed, we considered that there were no significant intraoperative spikes in the intraocular pressure.

Regarding the clinical features of visual acuity (VA) in PAMM, the mean VA was reported to range from 20/42 to 20/120.^11^ Generally, PAMM is said to have a good prognosis. However, the same literature reports that 50–72 % of eyes had VA ≥20/50, and 25–45 % of eyes had VA ≤20/200, indicating a variation in prognosis. Regarding visual prognosis, Nakashima et al. reported that 78 % of PAMM cases following vitrectomy for proliferative diabetic retinopathy improved to a VA of 20/40 or better.^6^ Our two patients also showed significant improvement in vision. However, caution is warranted, as there are reports where all 10 cases of PAMM following cataract surgery had a final VA worse than 6/60^5^, and there are instances where vision deteriorated to the level of light perception,^7^ indicating that the prognosis cannot be uniformly considered favorable. Paracentral scotomas often persist^1^^,^^5^^,^^10^; in our cases, the scotomas corresponded to the areas of INL thinning.

In the case of PAMM, where OCTA was performed at initial presentation, 3 days later, and 1 month later, a decrease in blood flow signals was reported at the middle capillary plexus and DCP levels initially. Partial improvement was noted after 3 days, and further improvement was observed after 1 month.^12^ Another report indicated that at the time of onset, blood flow signals were reduced in both the SCP and DCP levels, with improvements observed in the SCP level after 1 month, and mild residual vascular impairment at the DCP level but still improved.^13^ As indicated by these reports, decreased signals often improve within a few days to 1 month after onset. As highlighted in previous reports, a notable issue in PAMM is the thinning of the INL over time, which complicates the assessment of blood flow in the same layers. In our cases, although OCTA showed residual reductions in blood flow at the DCP level, these findings may not accurately reflect the true vascular status due to INL thinning. Sridhar et al. proposed three patterns in en face OCT of PAMM: arteriolar, fern-like, and globular.^14^ Each of these patterns is associated with mechanisms such as transient or true arterial occlusion, perivenular capillary ischemia, and distal precapillary or capillary ischemia. Our cases were considered to correspond with the arteriolar pattern, and the lesion in the inferior arcade may have resulted from local arterial occlusion. Occlusion may have occurred due to mechanical stimulation from the diamond-dusted membrane scraper used during ILM peeling in Case 1. There was no contact with surgical instruments in other retinal whitening areas, and the exact cause of the onset was unclear. However, for patients with atherosclerotic disease, it is advisable to minimize local anesthesia and reduce perfusion pressure as much as possible during surgery.

## 4 Conclusions

This report presented two cases of extramacular PAMM-like retinal ischemia after vitrectomy for ERM. Both patients were at risk of developing PAMM owing to anesthesia, intraoperative perfusion pressure, and systemic conditions that could lead to arteriosclerosis. However, blood flow disturbances at the DCP level persisted, along with associated visual field defects. Postoperative PAMM following ERM surgery is very rare, but is a complication that should be noted. Removal of ILM may result in deep retinal loss and should be included in the differential diagnosis of paracentral scotoma following ILM peeling.

## CRediT authorship contribution statement

**Chihiro Koiwa:** Writing – original draft, Data curation. **Pingyu Chi:** Investigation, Data curation. **Shutaro Yamamoto:** Supervision, Conceptualization. **Shintaro Nakao:** Writing – review & editing, Writing – original draft, Supervision, Conceptualization.

## Patient consent

Informed consent for the research and publication of this study and the accompanying images were obtained from the patient.

## Authorship

All authors attest that they meet the current ICMJE criteria for Authorship.

## Funding

Shintaro Nakao: Consulting fee by 10.13039/100010795Chugai Pharmaceutical, Kowa, 10.13039/100004336Novartis, Riverfield; travel reimbursements and speaker fees from 10.13039/100007816Alcon, Boehringer Ingelheim, Bayer Pharma, Canon, JFC Sales Plan, Kowa, Mitsubishi-Tanabe pharma, Machida, 10.13039/100030732MSD, 10.13039/100004336Novartis, 10.13039/100015758Novo Nordisk Pharma, 10.13039/100019120Otsuka, Santen Pharmaceutical, Senju Pharmaceutical, and 10.13039/501100004419Wakamoto Pharmaceutical. Chihiro Koiwa, Pingyu Chi, and Shutaro Yamamoto have no financial disclosures.

## Declaration of competing interest

The authors declare that they have no known competing financial interests or personal relationships that could have appeared to influence the work reported in this paper.
